# Population history in Okinawa based on JC virus and *ALDH2* genotypes

**DOI:** 10.1038/s41598-020-64194-y

**Published:** 2020-04-30

**Authors:** Daisuke Miyamori, Yuka Tanaka, Noboru Ishikawa, Tadaichi Kitamura, Hiroshi Ikegaya

**Affiliations:** 1 Department of Forensic Medicine, Graduate School of Medical Science, Kyoto Prefectural University of Medicine, Kyoto, 602-8566 Japan; 2 Department of Histology and Developmental Biology, Tokyo Dental College, Tokyo, 101-0061 Japan; 3 Shinsui Clinic, Tokyo, 132-0033 Japan; 4 Department of Urology, Graduate School of Medicine, The University of Tokyo, Tokyo, 113-8655 Japan

**Keywords:** Genotype, Genetic variation

## Abstract

It is widely known that people in Okinawa originated from the Jomon people, and are generally tolerant to alcohol. However, some individuals in mainland Japan lack alcohol tolerance due to a mutation in the human mitochondrial aldehyde dehydrogenase 2 (*ALDH2*) gene. Furthermore, the JC virus (JCV) genotype MY, which is related to the Jomon people, has not been found in Okinawa. In this study, to analyze the origin of the Okinawan people, we investigated the relationship between the JCV genotype and *ALDH2* genotype. We collected 108 JCV positive samples from Okinawa. Only CY genotype JCV, and not the MY genotype, was detected. Among JCV-positive samples, a variant of *ALDH2* (Glu/Lys heterozygote) was detected in 31 samples (29%) and wild-type *ALDH2* (Glu/Glu homozygote) was detected in 77 samples (71%). Another variant of *ALDH2* (Lys/Lys homozygote) was not detected. Among carriers of CY genotype JCV, wild-type *ALDH2* was much more frequent in people living in Okinawa than in mainland Japan (P < 0.05). Our results suggested that the original inhabitants of Okinawa were people who carried MY genotype JCV and wild-type *ALDH2*; and that after the extinction of these original inhabitants, people who carried CY genotype JCV and wild-type *ALDH2* migrated to the area. Due to the founder effect, CY genotype JCV and wild-type *ALDH2* became dominant. Over a long period, many people with the variant *ALDH2* migrated to Okinawa; the variant allele increased in frequency, but other JCV genotypes were eliminated.

## Introduction

Modern Japanese people are descendants of the Jomon people and Yayoi people. Archaeological studies have indicated that these two populations moved from the Chinese continent to the Japanese archipelago long ago. The Jomon people first migrated from the Chinese continent through the Korean peninsula about 37,000–38,000 BP^1^. The Jomon culture, who are known as hunter-gatherers and one of the older pottery cultures, then developed in 12,000 BP^2^. Long after the migration of the Jomon people, at 2,300 BP, the Yayoi people migrated from the Chinese continent to the Japanese archipelago. The migration of the Yayoi people resulted in the spread of the Jomon people to eastern Japan and the Okinawan islands (*dual structure model*)^3^. Genomic analyses of the ethnic origin of the Japanese population essentially support the *dual structure model*^4–7^.

In our previous study, we analyzed the relationship between mutations in the human mitochondrial aldehyde dehydrogenase 2 (*ALDH2*) gene and the JC virus (JCV) genotype to elucidate the ethnic origin of the Japanese population. Mitochondrial *ALDH2* is located on chromosome 12 and consists of 13 exons. The SNP rs671 in the *ALDH2* gene is a G-to-A substitution resulting in the ALDH2 Glu504Lys (originally, Glu487Lys) allele. This atypical *ALDH2* variant (ALDH2 Glu504Lys) is prevalent in Asians and is a causal factor in the alcohol flush reaction when drinking^8^. The variant of *ALDH2* has low levels of genetic diversity, suggesting a young haplotype^9^; it occurred in the Chinese Pai-Yuei tribe approximately 2,000–3,000 BP and gradually spread to peripheral regions^10^. Thus, it is considered a genetic marker for analyses of Yayoi ancestry^11^.

We investigated *human polyomavirus* JCV genotypes around the world^12^. *Homo sapiens* began to migrate from Africa about 100,000 years ago, with human parasitic JCV^13^. Since the migration, *H. sapiens* with JCV spread to many places in the world. Unlike in the human genome, integration does not occur in the viral genome, and therefore the minor genotype of the virus is not passed on to progeny, and does not persist in a group. Therefore, JCV genotypes are each distributed in a distinct area or population. This enables simple analyses of human migration. In a detailed analysis, Kitamura *et al.*^14^ found that MY genotype JCV is prevalent in eastern Japan and CY genotype JCV is prevalent in western Japan. Based on the substitution rate in the JCV genome, it is estimated that the MY clade arose 10,000–30,000 years ago^15^ and the CY clade arose 10,000 years ago. Therefore, archaeological findings combined with JCV evolution and distribution indicate that the Jomon people may be associated with MY genotype JCV and that the Yayoi people may be associated with CY genotype JCV.

We previously analyzed the relationship between JCV genotype and *ALDH2* genotype in people who live in mainland Japan. We found that people who carry CY genotype JCV have a higher frequency of *ALDH2* mutation than that of people who carry MY genotype JCV (P < 0.05)^16^. This result suggested that the Jomon people initially carried MY genotype JCV and wild-type *ALDH2* in the Japanese archipelago and, subsequently, Yayoi people who carried CY genotype JCV and a variant of *ALDH2* migrated from the Chinese continent throughout the Korean peninsula. The migration of the Yayoi people resulted in the eastward migration of the Jomon people towards eastern Japan. However, according to the dual structure model developed by archaeologists, the Jomon people were forced to move to the Okinawa islands as well as to eastern Japan. This model cannot explain why only CY genotype JCV is detected in Okinawa^14^. Therefore, in this study, we investigated *ALDH2* mutations associated with the Yayoi people among CY genotype JCV-positive people in Okinawa and discussed the origin of these populations.

## Results

In the 108 JCV positive samples, only CY genotype JCV was detected (see Supplementary Fig. S1). Among the JCV-positive samples, a variant of *ALDH2* (Glu/Lys heterozygote) was detected in 31 samples (29%) and wild-type *ALDH2* (Glu/Glu homozygote) was detected in 77 samples (71%). Another variant of *ALDH2* (Lys/Lys heterozygote) was not detected. Among people who carried CY genotype JCV, wild-type *ALDH2* was much more frequent in people living in Okinawa than in those in mainland Japan (Table 1) (P < 0.05).

**Table 1:** Frequency of the *ALDH2* variant among CY genotype JCV carriers in mainland Japan and Okinawa.

	Mainland Japan*	Okinawa
ALDH2 variant (Glu/Lys heterozygote)	17 (51.5%)	31 (29%)
ALDH2 wild type (Glu/Glu homozygote)	16 (48.5%)	77 (71%)
Total	33 (100%)	108 (100%)

Lys/Lys homozygotes were not detected in this study.

The difference in the number of *ALDH2* variants between mainland Japan and Okinawa was statistically significant (P = 0.027).

*From our previous study (Miyamori *et al.*^16^).

## Discussion

### Jomon and Yayoi people and the JCV genotype

MY and CY JCV are related to the Jomon and Yayoi people, respectively^14^. Wild-type *ALDH2* (Glu/Glu homozygote) and *ALDH2* Glu/Lys heterozygotes are associated with the Jomon and Yayoi people, respectively^11^. Therefore, it is estimated that genotypes MY and CY are associated with wild-type and variant *ALDH2*, respectively.

### People in mainland Japan

The MY clade of JCV arose 10,000–30,000 years ago^15^ and the CY clade of JCV occurred 10,000 years ago^17^. The Yayoi people migrated to the Japanese archipelago, where the Jomon people carrying MY genotype JCV lived about 2,000–3,000 years ago. The *ALDH2* variant arose about 2,000–3,000 years ago in the Chinese population^10^. The frequency of the *ALDH2* variant is 31.9% in the Japanese population^9^. However, among people living in mainland Japan who carry CY genotype JCV, the frequency of the *ALDH2* variant is 51.5%^16^. This frequency of the variant allele is much higher than that in the Chinese Guangdong Han (46.7%), and is the highest among Asian populations^9,10^. Therefore, when the Yayoi people first migrated to the western Japanese archipelago, they might have carried CY genotype JCV as well as the variant *ALDH2*. The group did not exchange genes immediately with the Jomon people, who carried MY genotype JCV and wild-type ALDH2; therefore, CY genotype JCV was not selected and the frequency of the *ALDH2* variant became high in this group. The Jomon people might have moved to eastern Japan and Okinawa according to the dual structure model. In eastern Japan, gene admixture occurred between the Jomon people and other groups with variant *ALDH2*, resulting in a gradual decrease in the frequency of the variant allele.

### People in Okinawa

The dual structure model predicts that the JCV genotype in Okinawa is MY. However, we only detected CY genotype JCV for all 100 JCV-positive samples, consistent with a previous report^14^. Additionally, the *ALDH2* variant was detected in 29% of people in Okinawa, but 51.5% in mainland Japanese (P < 0.05) (Table 1).

*ALDH2* mutations are related to drinking habits in the Japanese population^18,19^. Mutations at this locus are associated with hereditary diabetes^20^, risk factors for colon cancer^21^, esophageal melanosis^22^, hepatitis B virus infection^23^, and pancreatic cancer^24^. It is possible that these disease relationships influenced the mutation rate in the group.

Why was the frequency of the *ALDH2* variant among people with CY genotype JCV much lower in Okinawa than in mainland Japan? Additionally, why was CY the only genotype detected in Okinawa? We considered several explanations for these observations. JCV is inherited from either parent to offspring^25^. Minor JCV alleles were selected^26^. Therefore, populations included individuals who carried CY genotype JCV as well as MY genotype JCV with wild-type *ALDH2*. MY genotype JCV was lost over time. Only people who carried CY genotype JCV survived and spread to the Okinawan islands (bottleneck effect). However, this does not explain why only people who carried CY genotype JCV survived. There is little evidence for the Yayoi culture in Okinawa; accordingly, it is unlikely that CY genotype JCV pre-existed in the area. Instead, people in Okinawa might instead have carried wild-type *ALDH2*. When people who carried CY genotype JCV migrated from mainland Japan to Okinawa, CY type JCV spread in Okinawa via the founder effect. Some people who migrated from mainland Japan carried the *ALDH2* variant, and accordingly the frequency of the variant increased slightly in Okinawa by gene flow. However, considering that *Homo sapiens* migrated from Africa with JCV^27^, it is unlikely that the preexisting people in Okinawa did not carry JCV. In one scenario, Okinawa was uninhabited, and people from mainland Japan who carried CY genotype JCV and wild-type *ALDH2* migrated to the Okinawan islands by chance. By the founder effect, CY genotype JCV and wild-type *ALDH2* spread to in the Okinawan islands. Later, those who carried the variant *ALDH2* migrated to the Okinawan islands, and the variant genotype increased in frequency. Though the alternative JCV genotype might have been introduced more recently, the minor genotype was selected relative to the major CY genotype. Accordingly, even today, MY genotype JCV is not detected in Okinawa. However, there is some archeological evidence supporting human populations in Okinawa in ancient times. This contradicts the dual structure model. To explain the relationship between the JCV genotype distribution and the *ALDH2* variant in Okinawa, the following hypothesis was developed. People who carried MY genotype JCV and wild-type *ALDH2* existed in Okinawa. After the extinction of the alleles on the island, people who carried CY genotype JCV and wild-type *ALDH2* migrated. By the founder effect, CY genotype JCV and wild-type *ALDH2* became dominant. In the long history that followed, many people with the *ALDH2* variant migrated to Okinawa, resulting in an increase in the frequency of the allele. Although the alternative genotype of JCV might have been introduced to Okinawa, it was eliminated. In the history of Okinawa, a severe population decline occurred due to a tsunami and severe hunger caused by climate change. It is possible that early human populations were eradicated in Okinawa.

After the migration of the Yayoi people, gradual gene flow between the two populations might have occurred, influencing subsequent changes in the mutation rates of people in different areas in Japan. This is consistent with genomic comparisons among people living in Hokkaido, mainland Japan, and Okinawa^1,7,28^. However, the JCV genotype in a human population does not change easily^26,29^, it is ideal for population studies.

## Materials and Methods

### Materials

After obtaining informed consent, 50-mL urine samples were donated by 212 healthy volunteers who live in cities in Okinawa Island, Japan (Nago, Kita-Nakagusuku, Nakagusuku, Nishihara, Urazoe, Naha, Haebaru, and Tomigusuku). DNA samples extracted from urine were analyzed. The DNA extraction method was described by Kato *et al.*^26^. This study was approved by the Institutional Review Board of the Kyoto Prefectural University of Medicine (G-112). All methods were performed in accordance with the relevant guidelines and regulations.

### JCV detection and genotype classification

The 610-bp IG region^30^ that encompasses the 3′-terminal regions of both T antigen and *VP1* genes was PCR-amplified using the primers P1 and P2 and ExTaq Polymerase (TaKaRa Bio Inc., Kusatsu, Japan), as described by Kunitake *et al.*^25^. A total of 108 PCR-positive samples were used for genotype classification.

Amplified IG-region fragments were subjected to a cycle sequencing reaction using a BigDYE Terminator Cycle Sequencing Kit v. 3.1 (Applied Biosystems, Foster City, CA). Primers P1 and P2 were also used for sequencing, which was carried out with an automated DNA sequencer (3130 Genetic Analyzer, Applied Biosystems).

DNA sequences were aligned using the CLUSTALW program^31^, phylogenetic relationships among DNA sequences were evaluated using the neighborjoining (NJ) method^32^ via Kimura’s two-parameter distance method^33^. The phylogenetic tree was visualized using the NJ plot program^34^. To assess confidence levels within the phylogenetic tree, bootstrap probabilities (BPs) were estimated with 1,000 bootstrap replicates^35^. And finally, JCV genotype of each strain was confirmed.

### *ALDH2* genotype classification

A total of 108 JCV-positive samples were analyzed by real-time PCR, and the G-to-A substitution rs671 (i.e., the Glu504Lys or Glu487Lys polymorphism) in *ALDH2* was detected and classified. A fluorescence melting curve analysis was performed using a LightCycler (Roche Diagnostics GmbH, Mannheim, Germany) with primers and probes obtained from Takara Bio Inc. (TaKaRa Cycleave Human ALDH2 Typing Probe/Primer Set).

PCR conditions were as follows: 95 °C for 10 sec, followed by 60 cycles of denaturation at 95 °C for 5 sec, annealing at 53 °C for 10 sec, and extension at 72 °C for 20 sec. Fluorescence was measured during this process.

To confirm results of the above-mentioned method, we also analyzed the whole samples using the PCR-CTPP method as described^36^.

### Statistical analysis

The chi-square test for independence with Yates’ correction was utilized to compare the presence of the wild-type allele (i.e., normal *ALDH2* activity) in samples with JCV genotype CY collected in mainland Japan and Okinawa. A significant association between *ALDH2* genotypes and JCV genotypes was detected when P < 0.05. All analyses were performed using Microsoft Excel.

## Supplementary information

Figure S1.
