# Supplementary material for: Population history in Okinawa based on JC virus and ALDH2 genotypes
Source: Sci Rep. 2020 Apr 30;10:7331. doi: 10.1038/s41598-020-64194-y (PMC7192937; doi:10.1038/s41598-020-64194-y)
Supplement: Supplementary file 1 — Figure S1. [file 41598_2020_64194_MOESM1_ESM.pdf]

Population history in Okinawa based on JC virus and *ALDH2* genotypes

Daisuke Miyamori[1], Yuka Tanaka[1], Noboru Ishikawa[1,2], Tadaichi Kitamura[3] and Hiroshi Ikegaya[1]

1 Department of Forensic Medicine, Graduate School of Medical Science, Kyoto Prefectural University of Medicine, Kyoto 602-8566, Japan

2 Department of Histology and Developmental Biology, Tokyo Dental College, Tokyo 101-0061, Japan

3 Ashoka Hospital, Tokyo 135-0002, Japan

Corresponding author:

Hiroshi Ikegaya, MD, PhD

Department of Forensic Medicine, Graduate School of Medical Science, Kyoto Prefectural University of Medicine

465 Kajiicho, Kamigyo, Kyoto, Japan 602-8566

Ikegaya-tky@umin.ac.jp, ikegaya@koto.kpu-m.ac.jp

Running title: *ALDH2* and JC virus genotypes in Okinawa

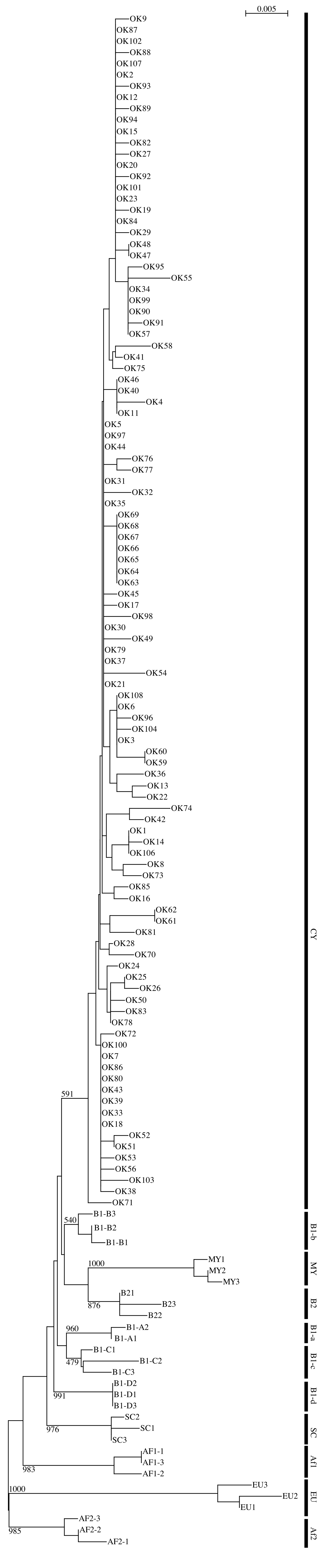

0.005
OK9
OK87
OK102
OK88
OK107
OK2
OK93
OK12
OK89
OK94
OK15
OK82
OK27
OK20
OK92
OK101
OK23
OK19
OK84
OK29
OK48
OK47
OK95
OK55
OK34
OK99
OK90
OK91
OK57
OK58
OK41
OK75
OK46
OK40
OK4
OK11
OK5
OK97
OK44
OK76
OK77
OK31
OK32
OK35
OK69
OK68
OK67
OK66
OK65
OK64
OK63
OK45
OK17
OK98
OK30
OK49
OK79
OK37
OK54
OK21
OK108
OK6
OK96
OK104
OK3
OK60
OK59
OK36
OK13
OK22
OK74
OK42
OK1
OK14
OK106
OK8
OK73
OK85
OK16
OK62
OK61
OK81
OK28
OK70
OK24
OK25
OK26
OK50
OK83
OK78
OK72
OK100
OK7
OK86
OK80
OK43
OK39
OK33
OK18
OK52
OK51
OK53
OK56
OK103
OK38
OK71
591
540
B1-B3
B1-B2
B1-B1
1000
MY1
MY2
MY3
876
B21
B23
B22
960
B1-A2
B1-A1
479
B1-C1
B1-C2
B1-C3
991
B1-D2
B1-D1
B1-D3
976
SC2
SC1
SC3
983
AF1-1
AF1-3
AF1-2
1000
EU3
EU2
EU1
985
AF2-3
AF2-2
AF2-1
CY
B1-b
MY
B2
B1-a
B1-c
B1-d
SC
Af1
EU
Af2

Figure legend

Figure S1. Phyologentic tree of JCV positive samples.
The phylogenetic tree was constructed from the IG sequences of 108 Okinawan samples by using the Neighbor Joining method. The phylogenetic tree was visualized by using the NJplot program.
